# Supplementary material for: Stacking Model‐Based Classifiers for Dealing With Multiple Sets of Noisy Labels
Source: Biom J. 2025 Mar 12;67(2):e70042. doi: 10.1002/bimj.70042 (PMC11898607; doi:10.1002/bimj.70042)
Supplement: Supplementary file 1 — Supporting Information [file BIMJ-67-e70042-s001.zip › density-based-ensemble-model-main/competitors_real_data/netlab-master/netlab-master/help/demgmm5.htm]

Netlab Reference Manual demgmm5


# demgmm5

## Purpose

Demonstrate density modelling with a PPCA mixture model.

## Synopsis

```
demgmm5
```

## Description

The problem consists of modelling data generated
by a mixture of three Gaussians in 2 dimensions with a mixture model
using full covariance matrices. The priors are 0.3, 0.5 and 0.2; the
centres are (2, 3.5), (0, 0) and (0,2); the variances are (0.16, 0.64)
axis aligned, (0.25, 1) rotated by 30 degrees and the identity
matrix. The first figure contains a scatter plot of the data.

A mixture model with three one-dimensional PPCA components is trained
using EM. The parameter vector is printed before training and after
training. The parameter vector consists of priors (the column), and
centres (given as (x, y) pairs as the next two columns).

The second figure is a 3 dimensional view of the density function,
while the third shows the axes of the 1-standard deviation ellipses
for the three components of the mixture model together with the one
standard deviation along the principal component of each mixture
model component.

## See Also

`gmm`, `gmminit`, `gmmem`, `gmmprob`, `ppca`

---

**Pages:**
Index


---

Copyright (c) Ian T Nabney (1996-9)
